# Plant dehydrins and dehydrin-like proteins: characterization and participation in abiotic stress response

**DOI:** 10.3389/fpls.2023.1213188

**Published:** 2023-07-06

**Authors:** Zofia Szlachtowska, Michał Rurek

**Affiliations:** Department of Molecular and Cellular Biology, Institute of Molecular Biology and Biotechnology, Faculty of Biology, Adam Mickiewicz University, Poznań, Poland

**Keywords:** dehydrins, dehydrin-like proteins, late embryogenesis abundant family, membranes, mitochondria, plastids, stress acclimation, unorganized structure

## Abstract

Abiotic stress has a significant impact on plant growth and development. It causes changes in the subcellular organelles, which, due to their stress sensitivity, can be affected. Cellular components involved in the abiotic stress response include dehydrins, widely distributed proteins forming a class II of late embryogenesis abundant protein family with characteristic properties including the presence of evolutionarily conserved sequence motifs (including lysine-rich K-segment, N-terminal Y-segment, and often phosphorylated S motif) and high hydrophilicity and disordered structure in the unbound state. Selected dehydrins and few poorly characterized dehydrin-like proteins participate in cellular stress acclimation and are also shown to interact with organelles. Through their functioning in stabilizing biological membranes and binding reactive oxygen species, dehydrins and dehydrin-like proteins contribute to the protection of fragile organellar structures under adverse conditions. Our review characterizes the participation of plant dehydrins and dehydrin-like proteins (including some organellar proteins) in plant acclimation to diverse abiotic stress conditions and summarizes recent updates on their structure (the identification of dehydrin less conserved motifs), classification (new proposed subclasses), tissue- and developmentally specific accumulation, and key cellular activities (including organellar protection under stress acclimation). Recent findings on the subcellular localization (with emphasis on the mitochondria and plastids) and prospective applications of dehydrins and dehydrin-like proteins in functional studies to alleviate the harmful stress consequences by means of plant genetic engineering and a genome editing strategy are also discussed.

## 1 Introduction

Abiotic stress threatens global plant functioning and results in secondary effects such as oxidative damage. Stress action is often related to suboptimal climatic changes that can hamper crop growth and production. Among the environmental stimuli that seriously affect plant productivity are drought, salinity, frost, cold, and heat (Sewelam et al., 2020; Bouabdelli et al., 2022). Abiotic stress leads to significant changes in various cellular compartments and at various physiological and molecular levels (Arora and Rowland, 2011; Rurek et al., 2018; Araújo et al., 2019; Yadav et al., 2023). Plant organelles, including mitochondria and chloroplasts, are particularly sensitive to stress, and alterations in organelle biogenesis have been observed under adverse conditions (Taylor et al., 2009). Within the cell response to stress, ROS produced by organelles, which are particularly dangerous to cellular integrity, can damage DNA and affect enzyme activity, thus modulating the metabolism (Sun and Lin, 2010). To properly maintain cellular homeostasis and organellar biogenesis, highly specific cellular and molecular responses and the coordination of diverse compartment functions in stress and recovery are indispensable (Welchen et al., 2014).

Dehydrins (DHNs), a class II protein of the late embryogenesis abundant protein family (LEA), are among the most important cellular players in the abiotic stress response. The disordered structure of DHNs allows them to be included in intrinsically disordered proteins (IDPs). Generally, in unbound form, DHNs lack defined secondary structures; only after binding to the ligand (e.g., the membrane lipid or metal ion) does their secondary structure helix appear, allowing those proteins to perform protective functions within the cell. In the last ten years, the flexibility and activities of DHNs were subjects of valuable literature reviews; however, some of these reviews focused mainly on diverse DHN functions (e.g., their involvement in membrane protection) and structural aspects rather than presenting more updated data on DHN involvement in stress response (Graether and Boddington, 2014; Liu et al., 2017; Murray and Graether, 2022).

This review provides an updated view of the segmental structure, tissue- and developmentally- specific accumulation, functions, and participation of various cellular DHNs in response to selected abiotic stress conditions. The discussed aspects are presented in the context of plant physiological and molecular alterations under stress acclimation, with an emphasis on interactions with organelles. Additionally, current opinions on the identity, subcellular localization of immunologically related dehydrin-like proteins (dlps), and their participation in the abiotic stress response are also presented. Finally, the lesser-studied points on DHNs and dlps and their future valuable applications for plant biology studies are discussed.

## 2 Dehydrin classification and sequence motifs

In the following chapter, we provide a general characterization of a class II protein from the LEA family. DHNs were initially identified in cotton (*Gossypium*) generative cells during embryogenesis and then in various plant species, including rice (*Oryza sativa*), barley (*Hordeum vulgare*), and maize (*Zea mays*) (Galau et al., 1986; Mundy and Chua, 1988; Close et al., 1989; Close, 1997). Later, they were found not only among other seed plants but also in liverworts (*Marchantiophyta*; Hellwege et al., 1994; Ghosh et al., 2016; Melgar and Zelada, 2021), mosses (*Bryophyta*; Saavedra et al., 2006; Ruibal et al., 2012; Agarwal et al., 2017), ferns (e.g., *Polypodium*; Layton et al., 2010), and even among *Cyanobacteria* (Close and Lammers, 1993). Fragmented and contradictory data exist on the presence of DHNs in algal taxa. The study by Li et al. (1998) suggests the presence of a protein immunologically related to DHNs of a broad size (17-105 kDa) in *Fucus*. These proteins appeared to be species- and developmentally specific and substantially thermolabile. Gasulla et al. (2009) immunodetected constitutively accumulated five proteins related to DHNs (ranging from 15 to 120 kDa in size) in the lichen chlorophytic photobiont *Trebouxia erici*. Becker et al. (2020) mention (without reference) a DHN in the green algae *Dunaliella salina*, which may be seen either as a result of a late evolutionary event or as an ancient feature lost during algae evolution. Unfortunately, none of these proteins were further characterized. On the contrary, Malik et al. (2017) could not find any algal DHNs despite an exhaustive genomic search. The striking feature of DHNs is their conformational dynamism, which is due to their primary structure and, particularly, the presence of numerous polar amino acid residues (Close, 1996). The polypeptide chain of DHNs does not contain tryptophan and cysteine, while more than 25% of their primary sequence is occupied by alanine or glycine (Close, 1997; Malik et al., 2017).

DHNs contain characteristic sequence motifs; their presence affects the functions and subcellular localization of DHNs. These proteins can be identified by their relatively high hydrophobicity and the presence of at least a single lysine-rich K-segment of 15 residues, which is typically C-terminal and contains up to 11 copies per protein (Close, 1997). Although the consensus sequence of the K-segment [XKXGXX(D/E)KIK(D/E)KXPG] was initially thought to be entirely conserved, recent studies have showed that the most conserved amino acids are in the motif core [(D/E)KIK(D/E)K], while the regions flanking the core are more variable (Malik et al., 2017). The amphipathic properties of the K-segment, with hydrophilic and hydrophobic residues, enable it to form secondary α-helical structures only in the presence of a ligand; otherwise, the protein is fully disordered (Close, 1996). Ligand interaction plays a vital role in providing the dynamic structure of DHNs (Koag et al., 2009). The properties of the K-segment are believed to be essential for DHN interactions with the other proteins (Liu et al., 2017; Hernández-Sánchez et al., 2019) and phospholipid membranes (Koag et al., 2009; Clarke et al., 2015) and to prevent protein aggregation (Drira et al., 2015; Szabała, 2023).

DHNs also include the Y- and S-segments. The N-terminal Y-segment [(V/T)D(E/Q)YGNP] often contains up to three tandem repeats. The C-terminal part of this motif, which is enriched with asparagine and glycine, is highly conserved, while aspartic acid at the N-terminus and proline at the C-terminus are also evolutionarily conserved (Close, 1997; Malik et al., 2017). The S-segment [LHR(S/T)GS_4–6_(S/D/E)(D/E)_3_], which contains serine repeats, mostly precedes the K-segment (Close, 1997; Malik et al., 2017). The S-segment is often phosphorylated, which influences the interaction of DHNs with Ca^2+^ and the localization of DHNs within the cell nucleus; it only partially regulates the nuclear localization (though not the dimerization) of selected DHNs, for example, wheat (*Triticum aestivum*) WZY1-2 protein (Alsheikh et al., 2003; Wang et al., 2020).

One of the less conserved DHN segments is the φ-segment. It contains polar and non-polar residues and is particularly enriched with glycine, glutamic acid, and threonine (Malik et al., 2017). The φ motif may be present in all DHN regions that are not within the conserved segment. Due to its relaxed secondary structure, it allows polar amino acids to interact with water molecules (Lv et al., 2018). Hughes and Graether (2011) have suggested that it is flexible and important for the maintenance of the DHN disordered structure, which would prevent interactions of partially denatured proteins.

The recently identified 18-residue-long F-segment, like the K-segment, contains hydrophobic residues within the core [EXXDRGXFDFX(G/K)] (Richard Strimbeck, 2017; Riley et al., 2019). It is present in different DHN subtypes, known from many genera, including *Rhododendron* and *Camelina* (Wei et al., 2019). For example, the F-segment was observed among numerous SK_n_ DHNs in ten proteins from the K_n_ subclass and in the SK_n_F subclass. The presence of the F-segment in the DHNs of gymnosperms and angiosperms suggests that it may be evolutionarily older than the Y-segment, which is found exclusively in angiosperms (Riley et al., 2019). The presence of the F-segment (and the K-, Y-, and S-segments) has also been confirmed among halophyte DHNs. Interestingly, the role of DHNs in salinity adaptation is poorly studied (Ghanmi et al., 2022). It has been suggested that the F-segment may play a role in the membrane and in protein binding (Richard Strimbeck, 2017); some recent findings by Ohkubo et al. (2020) have provided additional information on its cryoprotective properties.

Riley et al. (2019) mentioned another DHN motif consisting of a stretch of consecutive lysine residues separated by arginine, aspartic acid, and glutamic acid; it occurs frequently between the S and K motifs. This motif was observed only among K_n_S and SK_n_ DHNs and in a single DHN from the K_n_ subclass. Other potential motifs are still described. Another newly identified segment was recently discovered only in the moss *Syntrichia ruralis*. It is closely related to the Y-segment and similarly to the F- and K-segments. It contains hydrophobic residues, which enable the formation of amphipathic α-helices. Their properties, which allow the self-association of DHNs, are indispensable for stress tolerance (Upadhyaya et al., 2021). Moreover, Yokoyama et al. (2020) noted ‘non-K segment 1’ in Arabidopsis HIRD11 DHN. The analysis of various plant genomes containing genes for K_n_S DHNs revealed the presence of an N-terminal motif consisting of 15 residues (the H-segment). This motif was identified in angiosperms, gymnosperms, and lycophytes, indicating the ancestral origin of this subclass, potentially tracing back to the early stages of land plant evolution (Melgar and Zelada, 2021). In addition to the mentioned motifs, DHNs contain other repetitive fragments that affect their properties, such as histidine-rich sequences that affect the ability of LEA-2 proteins to bind ligands such as metals (Hara et al., 2005; Hara et al., 2016) and lipids (Eriksson et al., 2011) and to interact with the other DHNs (Hernández-Sánchez et al., 2014; Hernández-Sánchez et al., 2017). As will be indicated below, histidine-rich sequences also increase membrane affinity (Eriksson et al., 2011; Gupta et al., 2019).

**Figure 1:**
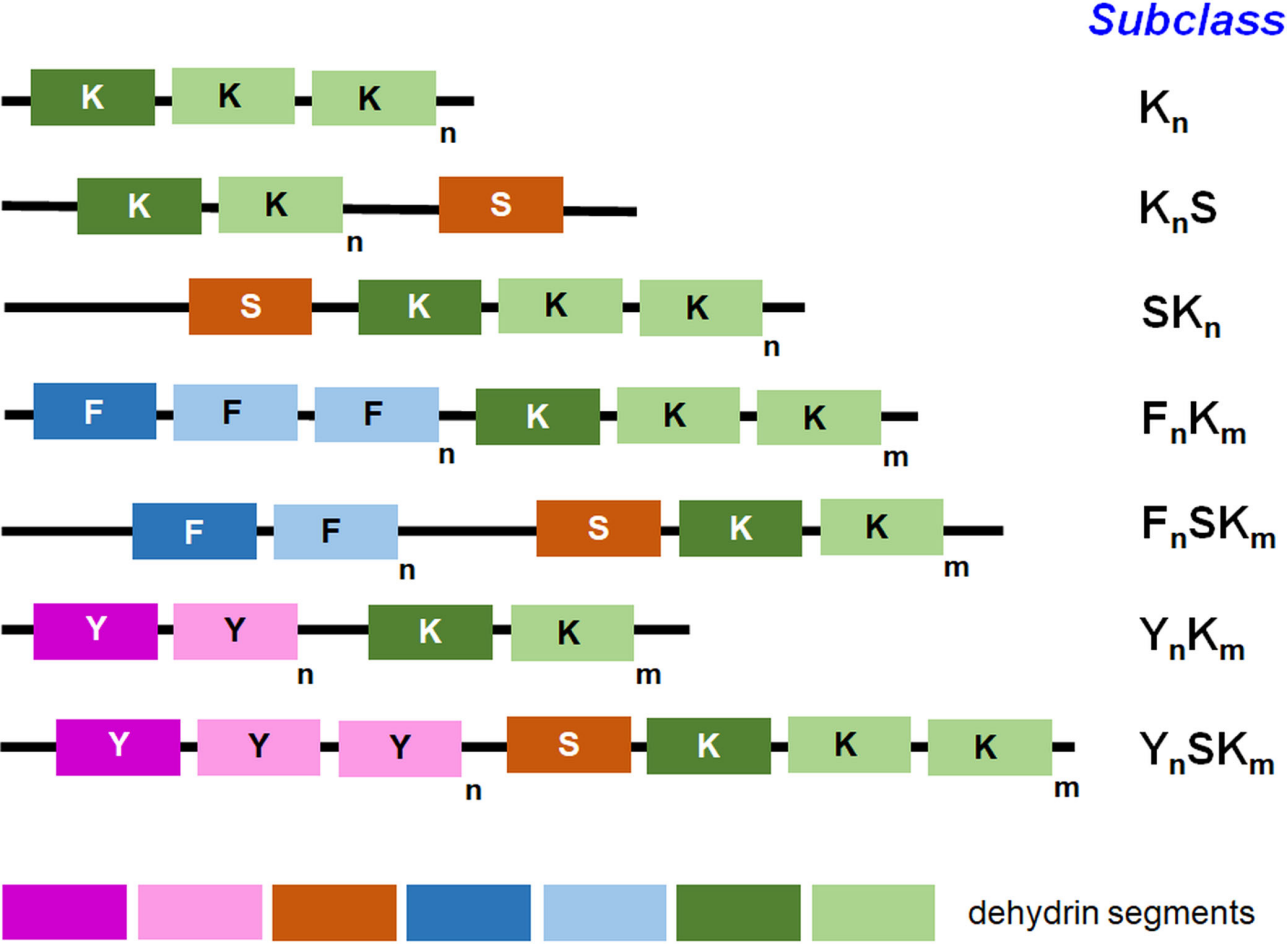
Diverse dehydrin subclasses based on the segment presence. The key segments and their order in each subclass are shown. Among others, the K-, S-, Y-, and F-segments belong to the well-known ones; ‘m’ and ‘n’ letters in the subscript (next to small rectangles depicting protein segments) indicate the number of a given segment in each subclass. The number of repeats for particular segments that are presented in the figure by various color tones is just exemplary and reflects the diversity in the segment number across various subclasses. The distance between the drawn segments is shown highly schematically and does not reflect the real distance between the segments in the particular dehydrin molecule.

Overall, based on the arrangement of domains and their number, the subtypes K_n_, SK_n_, K_n_S, Y_n_K_n_, Y_n_SK_n_, F_n_K_n_, and F_n_SK_n_ DHN (n denotes the number of copies of a given segment; Richard Strimbeck, 2017; Riley et al., 2019; Upadhyaya et al., 2021; **Figure 1**) can be assigned to three orthologous DHN clusters containing H-, F- or Y-segments (Melgar and Zelada, 2020).

The chemical properties of amino acid residues affect the structure of proteins. Hydrophobic amino acids present in the core of the chain allow globular proteins to fold properly. On the contrary, DHNs contain an excess of polar residues that impact their hydrophobicity (Close, 1997), resulting in the disorganization of the polypeptide chain (Tompa, 2002; Tompa et al., 2005).

Intrinsically disordered proteins (IDPs), including DHNs, do not have a fixed three-dimensional structure. Because of their lack of stable structure, IDPs retain their inherent integrity even in unfavorable conditions, which makes them incapable of denaturation. In general, they can dynamically change their structure. For this reason, it is not the rigid, stable, and fixed protein structure that determines the functions of IDPs. Rather, their role is mainly based on subcellular localization and sequence motifs (Tompa et al., 2005; Tompa et al., 2006). The structural plasticity of IDPs allows them to perform various cellular functions. For example, the binding of different ligands within a region or changing the DHN conformation depends on current cellular conditions and thus alters the functions performed, which is referred to as ‘moonlighting’ (Tompa et al., 2005). As a result of the association with phospholipid-containing vesicles, DHNs can acquire a helical structure (Koag et al., 2003) in which hydrophobic residues are located on one side and hydrophilic residues are located on the other side of the polypeptide chain (Eriksson et al., 2016). However, under stressful conditions, DHNs may still be in a disordered state, which is only slightly affected by solvent alterations. DHNs maintain such a disordered structure in stress, contrary to other proteins in which the structural collapse of the unfolding state has often been observed (Mouillon et al., 2008).

## 3 Tissue- and developmentally-specific dehydrin accumulation

DHNs are most often localized in young, still-growing, and differentiating tissues such as root cells, young stems or petioles, root meristems, phloem, pollen sacs, and germ cells (Nylander et al., 2001; Rorat, 2006). Changes in DHN accumulation under stress are not exclusive to generative tissues but also occur in vegetative organs (Close, 1997). DHN accumulation is usually induced by various adverse conditions, such as cold, drought, or frost, which affect gene expression and post-translational modifications of DHNs (Sun et al., 2021b). The level of some DHNs can be elevated with stress. Examples include LTI29 and ERD14 in Arabidopsis, which are present in the root apex, root vascular tissues, and shoots, or the RAB18 protein, localized in the protective gurd cells of the stomata apparatus (Nylander et al., 2001).

However, multiple genes and proteins play a role in the vegetative/generative phase transition (Yadav et al., 2023), and it is also expected that DHNs will participate in this tightly regulated and complex process. For example, Nazemof et al. (2016) identified EDR14 DHN (often annotated as a pollen coat protein) in the *Brassica napus* stigma proteome (ca. 2700 proteins detected). Furthermore, investigation of the proteome of *B. rapa* floral buds at meiosis, which allowed a better understanding of the relevance of meiosis and polyploidization events in vegetative/generative transition, revealed the downregulation of the ERD14 protein in the autotetraploid vs. diploid line (Yang et al., 2019a).

DHNs belong to numerous desiccation-tolerance proteins identified in plant seeds; furthermore, other LEA, seed maturation, and embryonic maturation proteins participate in seed maturation and imbibition. However, the expression of DHNs in mature seeds and tissues (e.g., leaves) under drought relies on common molecular mechanisms induced during water shortage; moreover, differences in DHN abundance among diverse seed types should be assigned to the maturation drying (Radwan et al., 2014). The DHN level may differ in *B. napus* lines with a high or low oil content (Gan et al., 2013; Gu et al., 2016). For example, Rahman et al. (2021) identified Rab18-like and Xero1-like DHNs in the *B. rapa* seed proteome, and Gan et al. (2013) showed a markedly increased level of Rab18 in the rapeseed low-oil-containing genotype; however, few LEA proteins appeared to be more expressed in the line with high oil content. Rihan et al. (2017) analyzed, inter alia, the temporal profiles of DHNs in cauliflower (*B. oleracea* var. *botrytis*) developing seeds. Proteins of 12, 17, and 26 kDa representing small and medium-sized DHNs increased in abundance 60 days after pollination.

Legume DHNs enriched with the Y-segment accumulate primarily in seeds (Mota et al., 2019). The DHN present in pea (*Pisum sativum*), known as DHN-COG, accumulates during plant embryogenesis in the developing cotyledons of seedlings under water-deficient conditions. As the embryo matures, DHN-COG accounts for approximately 2% of all seed proteins (Robertson and Chandler, 1994). The Arabidopsis DHN protein LTI130 also accumulates under stress conditions and is expressed in vegetative tissues only under cold conditions (Nylander et al., 2001). In turn, Arabidopsis ERD14 and ERD10 proteins under control conditions of plant growth and development are found mainly in stem tissues, leaves, flowers, and root cells, but under cold stress they are detectable in all studied plant tissues (Abdul Aziz et al., 2021).

## 4 Interactions of dehydrins with membranes and organelles

Lipid membranes allow the functional compartmentalization of various metabolic processes within the membrane continuum: cell membrane—inner membranes and vesicles—nuclear envelope. Membranes are particularly sensitive to temperature fluctuations and dehydration, which cause changes in the lipid structure and composition. In addition, low temperature causes membrane lipid peroxidation, an elevated formation of reactive oxygen species (ROS), and a reversible transition from a lamellar to a hexagonal membrane, which affects membrane functions (Thomashow, 1999). In contrast, as the temperature increases, the content of unsaturated fatty acids within the lipid membranes decreases (Falcone et al., 2004), resulting in reduced fluidity and increased membrane rigidity.

DHNs are not only cytoplasmic proteins; they also interact with various cellular membranes, including plasma, nuclear, and tonoplast membranes (Houde et al., 1995; Danyluk et al., 1998; Heyen et al., 2002; Zhang et al., 2020), and the physicochemical effects of such interactions have been subject to various studies. Although plant DHNs are localized primarily in the cytoplasm and nucleus, they can also be found near the cell membrane, mitochondria, and plastids due to their involvement in protecting these organelles (Candat et al., 2014; Kalemba et al., 2015). The effects of the binding of DHN to membranes on the secondary structure of DHNs were investigated using dodecylate-containing micelles; the circular dichroism (CD) revealed changes in the structure of the studied DHNs and the presence of α-helical structures (Ismail et al., 1999).

An essential factor in the interaction between DHNs and membranes is the positively charged K-segment, which facilitates the interaction of DHNs with negatively charged membranes (Koag et al., 2009). However, it should be underlined that not only the K- or Y-segment properties are crucial for the DHN-membrane binding. The cellular functioning of DHNs also depends on the composition of less conserved regions rich in polar and charged residues. In addition, post-translational modifications also affect DHN biological activity; all these factors can differentiate various DHNs even from the same species (Vazquez-Hernandez et al., 2021). LTI30 is a DHN that contains six K-segments, five of which are flanked by histidine-rich sequences. When the helical conformation occurs within the K-segment, the structure of LTI30 changes, allowing the histidine-rich regions to interact with the lipid ‘heads’ (Eriksson et al., 2016; Gupta et al., 2019; Andersson et al., 2020). Lipid phase transitions significantly affect membrane function. The LTI30 protein has been shown to reduce the temperature of the liposome phase transitions, allowing membrane fluidity to be maintained at reduced temperatures (Eriksson et al., 2011). Studies of the binding of Arabidopsis ERD10 and ERD14 to liposomes have shown that they mainly interact with lipid ‘heads’ of the membrane surface and do not directly affect membrane fluidity (Kovacs et al., 2008). However, some domains engaged in partner binding and other regions of unstructured ERD14 are indispensable, to a diverse extent, for its functioning (Murvai et al., 2021).

The way in which DHNs interact with lipid membranes (also organellar ones) is primarily determined by the membrane content. Phosphatidylcholine (PC) is the most prevalent lipid in plant membranes, while phosphatidic acid (PA) is less common, accounting for only 1-2% of membrane compounds (Graether and Boddington, 2014). DHNs exhibit the strongest affinity for PA and the weakest affinity for PC (Koag et al., 2003), which becomes significant under stress conditions where changes occur in the lipid content of the membrane. During stress, such as water deficiency, the PA content of the membrane increases, enabling DHN to bind and protect membranes from damage (Munnik, 2001; Koag et al., 2003). The diversity of lipids that build biological membranes is relevant for the specific organelles with which DHNs interact. For example, an experiment analyzing the conformation of the *Ts*DHN-1 dehydrin of *Thellungiella salsuginea* suggested that it can acquire the β-sheet structure when it interacts with the membrane lipids. The study used membranes that mimic the properties of organellar membranes, which are mainly composed of monogalactosyl- (MGDG) and digalactosyl-diacylglycerols (DGDG), two neutral galactolipids that differ from other cellular membranes (Rahman et al., 2011; Rahman et al., 2013).

To ensure proper organellar biogenesis, it is vital to maintain membrane stability and permeability. Nagaraju et al. (2019) analyzed *in silico* sorghum (*Sorghum bicolor*) DHNs and predicted plastid targeting for nearly 60% of these proteins. Using GFP fusions, Li et al. (2017) experimentally showed that the *Pp*DHNC protein from *Physcomitrella patens* is targeted to plastids and suggested that it may help stabilize thylakoid and other plastid membranes while maintaining electron flow between photosystems. COR15a and COR15b are also among the DHNs that interact with plastid membranes in cold and will be discussed in the following paragraphs (Thalhammer et al., 2014).

DHN and dehydrin-like proteins (dlps; recognized with antisera against the K-segment but more thermolabile than DHNs; see Chapter 7 for more details) have also been found to interact with mitochondrial membranes. An example is the *Citrus unshiu Cu*COR15 protein, whose subcellular localization was examined by the centrifugation fractionation; it likely binds to the mitochondrial membrane and inhibits lipid peroxidation (Hara et al., 2003). This is particularly important because mitochondrial membranes are sensitive to ROS. When studying the impact of drought on the biogenesis of cauliflower (*B. oleracea* var. *botrytis*) mitochondria, an increase in the expression level of the ERD14 gene that encodes DHN interacting with membranes of purified mitochondria was observed under water deprivation (Rurek et al., 2018). Borovskii et al. (2005) investigated whether proteins detected by K-segment-specific antisera bind to the outer mitochondrial membrane and if they can be imported into the mitochondrial matrix or bind to the inner membrane. Their findings revealed that certain thermostable DHNs interact with the outer mitochondrial membrane and do not penetrate the organelle. Meanwhile, Romanenko et al. (2010) observed DHNs in wheat seedlings near both the inner and outer mitochondrial membranes. Szabala et al. (2014) used immunogold labeling to indicate the precise binding site of DHN24, and their study of the subcellular targeting of DHNs of the SK_3_ subclass demonstrated that this DHN binds solely to the outer mitochondrial membrane on the cytosolic side but does not undergo translocation into the matrix.

## 5 Dynamic pattern of cellular and organellar dehydrins in abiotic stress responses

Many stress stimuli, for example, temperature treatments, also result in developmental delays, reduced membrane fluidity, and even cell death, as well as in the protein biosynthesis, production of solutes, and altered plant morphological, physiological, biochemical, and molecular responses. In numerous plant species, proteomic adjustments to abiotic stress also cover defense and protective proteins (Yadav et al., 2023); the level of these proteins affects the tolerance of the plant to a given stress stimuli, thus increasing stress acclimation (Arumingtyas et al., 2013; Graether and Boddington, 2014). Together with antioxidant proteins, metabolism enzymes, and heat shock proteins (HSPs), DHNs belong to proteins whose abundance is significantly modulated under stress (Schlesinger, 1990; Tamburino et al., 2017). DHNs have been noted in various cellular compartments, including plastids and mitochondria, under a multitude of adverse conditions (NDong et al., 2002; Mueller et al., 2003; Grelet et al., 2005; Hundertmark and Hincha, 2008). Some DHNs (e.g., DHN1 from *Arachis duranensis*) are involved in contradictory responses from various stress stimuli and can increase plant tolerance only to the particular treatment (Mota et al., 2019). The data below will summarize recent advancements in elucidating the participation of various cellular DHNs (including organellar proteins) in responses to temperature and drought stress. **Table 1** also presents a summary of selected organellar DHNs (and the immunologically related dlps) whose accumulation increased or was induced in response to various abiotic stress conditions.

**Table 1:** Alterations in the level of selected plant organellar dehydrins (DHNs) and dehydrin-like proteins (dlps) under abiotic stress.

Dehydrins (or dlps)	Subcellular localization	Stressor	Species	Reference
50-70 kDa dehydrins, 10-100 kDa dlps (*Ath*, *Bol*), 25-50 kDa dlps (*Ll*)	outer mitochondrial membrane, mitochondrial matrix	cold, freeze, heat	yellow lupin (*Lupinus luteus*, *Ll*)	Rurek, 2010
			cauliflower (*Brassica oleracea* var. *botrytis*, *Bol*)	
			*Arabidopsis thaliana* (*Ath*)	
52 kDa, 63kDa, 196 kDa and 209 kDa dehydrins, 28-63 kDa dlps	mitochondria	cold, freeze, drought, ABA treatment	wheat (*Triticum aestivum*)	Borovskii et al., 2002; Borovskii et al., 2005
			rye (*Secale cereale*)	
			maize (*Zea mays*)	
DHN24	outer mitochondrial membrane, cytoplasm, nucleus	cold, drought	*Solanum sogarandinum*	Szabala et al., 2014
COR15	mitochondria	cold	satsuma orange (*Citrus unshiu*)	Hara et al., 2003
31kDa and 37 kDa DHNs	chloroplasts	drought	pea (*Pisum sativum*)	Mueller et al., 2003
*Pp*DHNA, *Pp*DHNC	chloroplasts	cold, salinity	*Physcomitrella patens*	Li et al., 2017
SbLEA	chloroplasts, cytoplasm, nucleus, mitochondria	cold, heat	sorghum (*Sorghum bicolor*)	Nagaraju et al., 2019
PCA60	cytoplasm, nucleus, chloroplasts	cold	peach (*Prunus persica*)	Wisniewski et al., 1999
COR15a	chloroplasts	cold, freeze	*Arabidopsis thaliana*	Artus et al., 1996; Thalhammer et al., 2014
COR15b	chloroplasts	cold	*Arabidopsis thaliana*	Wilhelm and Thomashow, 1993

### 5.1 Cold and frost

Cold and freeze exert multilevel effects on plant organisms; in numerous crops, they share a plethora of specific and common responses (Raza et al., 2022). The cold acclimation process has been well studied and involves the accumulation of various DHNs that positively affect the cold hardiness of plants. For instance, a study by Artus et al. (1996) showed that the accumulation of DHNs increased the cold hardiness of Arabidopsis by about two degrees. Kosová et al. (2014) demonstrated that different DHNs accumulated at different cell sites of common wheat (*T. aestivum*) and barley (*H. vulgare*) under various stress conditions, including low-temperature treatments. Similarly, Rorat et al. (2006) observed an increase in the DHN24 level in stress-tolerant *Solanum* species after cold treatment, while no significant changes were observed in low-temperature-sensitive species. Cold-tolerant genotypes of barley and wheat accumulated more DHNs at a faster rate than sensitive lines (Vítámvás et al., 2019). Another DHN protein, WSCOR410, was found to promote cold tolerance in wheat (Danyluk et al., 1998).

Interestingly, grass proteins mostly from the K_n_, SK_n_, and K_n_S types appear to be regulated by low temperature; acidic SK_3_ DHNs, including the WCOR410 family in common wheat (*T. aestivum*) and small KS DHNs, are predominantly cold induced, sometimes in the generative tissues (Danyluk et al., 1998; Choi et al., 1999; Rodríguez et al., 2005; Wang et al., 2014). Few structural DHN subtypes (including K_n_, FSK_n_, SK_n_, Y_n_SK_M_, and K_n_S) have been detected in wheat (*T. aestivum*) and barley (*H. vulgare*) (Kosová et al., 2014; Richard Strimbeck, 2017). The Y_n_SK_m_ subfamily not only seems to be the most abundantly represented in those species, but it also contains cytoplasmic and nuclear proteins, which are mainly induced by dehydration stress and under ABA, MeJA, SA, or GA treatments. Furthermore, K_n_-type abundance has been shown to be regulated by cold, drought, and ABA treatment, e.g., WCS120 and WDHN13 in common wheat (Choi et al., 1999; Wang et al., 2014). The distinct DHN subtype, FSK_n_, is represented by cold-inducible DHNs of *Rhododendron catawbiense*; for example, the abundance of *RcDhn1-5* transcripts increased vastly in cold and decreased rapidly under stress deacclimation (Wei et al., 2019). Moreover, it has been demonstrated that *in planta* overexpressed *Capsicum annum* DHN4 protected Arabidopsis plants against the negative consequences of cold (Zhang et al., 2020).

The mechanism behind cold acclimation involves membrane protection by DHNs. For example, *Si*DHN from *Saussurea involucrata* protects leaf plastids from cold and drought; its maximal accumulation was observed after two days of cold treatment (Guo et al., 2019). La Porta et al. (2015) found that many mRNAs encoding DHNs were differentially expressed during cold acclimation in Italian cypress (*Cupressus sempervirens*), suggesting that DHNs play a role in protecting cells and plastid membranes from cold.

Data on the participation of DHNs in cold acclimation are particularly broad for the *Brassica* genus. Recently, Niu et al. (2021), using a *non-gel* approach, investigated the proteomic profiles of two field-grown winter rapeseed cultivars (*B. rapa*) that shared diverse sensitivity to freezing temperatures (-11°C). They showed alterations in the abundance of various DHNs in frost. In the cold-tolerant genotype ‘*Longyou 7*’, two DHNs were upregulated (Rab18-like most highly and ERD10 to a lesser extent); however, in the stress-sensitive cultivar ‘*Lenox*’, two additional proteins appeared moderately upregulated, ERD14 and HIRD11. Here, more DHNs assisted in stress adaptation among stress-sensitive genotypes. When two other winter rapeseed cultivars with contrasting frost sensitivity were treated in different freezing conditions (-4°C) by Wei et al. (2021), the results were only partially similar to the study by Niu et al. (2021). Interestingly, in the experiment of Wei et al. (2021), additional DHN isoforms (ERD14-like isoform X2 and Rab18-like isoform X1) were upregulated, and Xero 2-like DHN declined strikingly in abundance between both investigated cultivars. Furthermore, Jankauskienė et al. (2022) proposed the participation of thermostable proteins of a broad size (10-150 kDa), including DHN, in the development of winter tolerance of the cold-resistant ‘*Valesca*’ rapeseed (*B. napus*); in this genotype, DHNs of 37-65 kDa were identified in autumn growth, and the small DHN (25 kDa) accumulated only at the end of the cold acclimation period. In general, DHNs of 25-47 kDa should be recognized as potential candidates for cold acclimation markers in rapeseed.

COR15a and COR15b are plastid DHNs that accumulate in response to various stress stimuli. However, COR15b, which shares up to 82% of the primary sequence with its homologue, COR15a, accumulates only under cold and ABA treatments and not under water deficiency. Analysis of transcripts for both proteins revealed that *cor15b* messengers accumulate primarily under cold and ABA treatment (Wilhelm and Thomashow, 1993). It should be added that both COR15 proteins protect the plastid membranes from damage during cold treatment, which will be further discussed in Chapter 6. This protective role of DHNs reflects the relevance of the plastid in the development of cold tolerance (Maryan et al., 2021). In particular, the level of expression of the *COR15a* gene in the cold can be further stimulated by exogenous melatonin treatment, suggesting that upregulation of cold-responsive genes by melatonin may enhance the synthesis of cold-protective molecules that substantially alleviate transgenic plant growth in decreased temperatures. Melatonin, a very notable ‘defense compound’, protects plants from the effects of temperature stress by affecting stress defense mechanisms and interacting with phytohormones and a variety of other molecules. Therefore, it plays a key role in the regulation of the temperature response, acting synergistically or antagonistically with multiple compounds across signaling pathways (Bajwa et al., 2014; Raza et al., 2022).

Mitochondria-associated DHNs are also involved in cold acclimation. *In vitro* studies have revealed that *Thellungiella salsuginea* DHNs (*Ts*DHN1 and *Ts*DHN-2) interact with liposomes that mimic mitochondrial membranes, but their mechanisms of interaction are different, even though both proteins bind to the same membranes (Rahman et al., 2013). Studies on changes in the accumulation of DHN in tobacco (*Nicotiana tabacum*) expressing one of the *Citrus* DHN (predominantly expressed in mitochondria) confirmed that the expression of this protein increased the resistance of tobacco plants to reduced temperature, accompanied by earlier seed germination and inhibited lipid peroxidation (Hara et al., 2003).

The contribution of DHNs to cold deacclimation has been observed in two rose (*Rosa*) garden cultivars that differed in their sensitivity to low temperature: *‘Dagmar Hastrup*’ and ‘*Chandos Beauty*’. In the cultivar with higher tolerance to cold, more *Rh*DHN5 accumulated during cold exposure, and as the temperature increased, the levels of DHN were further altered (Ouyang et al., 2020). Similar results were obtained for DHNs of blueberry (*Vaccinium*) and peach (*Prunus persica*) (Arora and Rowland, 2011).

It should be added that the expression of *LEA/DNH* genes under lowered temperatures (and under selected other environmental conditions) is governed, inter alia, by dehydration-responsive element-binding protein (DREB)/CBF transcription factors (belonging to plant-specific APETALA2/ethylene-responsive element-binding factors). For example, in Arabidopsis plants that overexpress the DREB1 protein under the strong promoter, the over-representation of *LEA*/*DNH* genes among upregulated genes was notable under the action of these unfavorable stimuli but not under heat stress (Kidokoro et al., 2015). The expression of the maize (*Z. mays*) cold-induced DHN15 protein is governed by *cis* elements related to the cold response. DHN15 overexpression has been shown to significantly improve yeast cold resistance, and *in planta* overexpression also resulted in the improvement of numerous biochemical, physiological, and molecular stress markers (Chen et al., 2023).

The DHN interactome in cold stress involves key signaling transducers. For example, Liu et al. (2019) showed that WZY2 DHN interacts with protein phosphatase 2C to regulate cold and osmotic stress-responsive genes in wheat, shedding new light on the regulatory mechanisms that govern DHN expression.

### 5.2 Heat stress and interactions with other stress conditions

Heat treatment is one of the most critical environmental stimuli for plant growth and productivity; it may lead to pollen sterility, impaired seed development, and other aberrations (Yadav et al., 2023). It accelerates senescence and disturbs numerous physiological processes, including photosynthesis and respiration, and it also enhances ROS production and leads to perturbations in carbon skeleton metabolism. The increase in DNHs synthesis under heat stress should be regarded as a part of more complex molecular mechanisms, including the production of heat-protective compounds in plant cells (e.g., HSPs; Raza et al., 2022).

Zhou et al. (2017) investigated the expression of the cucumber *CsLEA11* gene (*Cucumis sativus*) in the *E. coli* strain under heat, resulting in an increase in the abundance of *CsLEA11* transcripts positively correlated with the gain of heat tolerance. Furthermore, the accumulation of the *Cs*LEA11 protein allowed the maintenance of the *in vitro* activity of the bacterial lactate dehydrogenase (LDH); Halder et al. (2017) obtained similar results for the DHN1 protein. In the Wahid and Close (2007) study, sugar cane (*Saccharum officinarum*) seedlings were exposed to different conditions of heat treatment (45 or 35°C). The elevated temperature resulted in the accumulation of three distinct DHNs, improving the tolerance to plant stress by maintaining membrane integrity. Transcripts for another dehydrin (DHN8) and other LEA proteins were notably induced in the stress-sensitive ‘*Golden Promis*e’ barley cultivar (*H. vulgare*) under high-temperature treatment (Mahalingam et al., 2022). In the newly investigated *Brassica campestris* genotype, the upregulation of two diverse ERD DHNs (ERD10 and ERD14) for both low- and high-temperature treatments was notable (Yuan et al., 2019). On the contrary, in lettuce (*Lactuca sativa*), other proteins of the same family (ERD15) decreased in abundance under heat (Hao et al., 2018).

It should be emphasized that the impact of heat treatment on the expression pattern of *DHN* genes was often studied in combination with other stimuli. Sunflower (*Helianthus annuus*) DHN expression was assayed by Abdel Razik et al. (2021) under heat alone and under heat combined with the γ-aminobutyric acid (GABA) treatment. GABA treatment resulted in increased accumulation of DHNs, and the interaction of heat and GABA treatments caused an additional increase in the level of DHNs; this allowed maintaining such a high level of DHNs in the following days. Interestingly, the accumulation of olive (*Olea europaea*) *DHN1* transcripts was increased by the heat treatment and additionally by the water shortage; such studies appear to be important for the cultivation of this species, often grown in insolated and water-deficient areas (Araújo et al., 2019). Furthermore, Hao et al. (2022) compared the expression of *DHN* genes in wheat (*T. aestivum*) in heat, cold, and drought. Although almost 80% of *DHN* genes appeared to be expressed under those conditions, *DHN1-3* genes were quite weak; heat affected the expression pattern of *DHN* genes less effectively (5-8 genes under various heat treatments). On the contrary, more wheat *DHN* genes became highly expressed under cold and drought (compare with Chapters 5.1 and 5.3).

Analysis of the accumulation of DHNs and dlps under the influence of temperature stress (increased or decreased temperature, including cold and frost) revealed a stress-induced increase in the number of these proteins associated with the mitochondria of cauliflower (*B. oleracea* var. *botrytis*), Arabidopsis, and yellow lupin (*Lupinus luteus*) and a simultaneous increase in their accumulation. This increase varied between the individual studied species—notably, in cauliflower and Arabidopsis—and the stress response included more significant differences in the level of various-sized DHNs and dlps. In contrast, in lupin mitochondria, alterations in the level of DHNs/dlps were less pronounced at unfavorable temperatures (Rurek, 2010).

### 5.3 Water deficit

Long-term and frequent drought treatments, which contribute to soil water shortages, pose a great threat to crops. Frequently, they accompany heat stress, affecting seed germination, plant growth, and the transition from the vegetative phase to the generative phase, as well as decreasing yield and seed quality. The adaptation to drought is a very complex process, altering the expression level of numerous genes; additionally, the level of ABA and protective solutes is affected, antioxidative pathways are induced, and energy-consuming pathways are inhibited (Salekdeh et al., 2009). The DHN pattern under water deficit can be particularly complex and tissue specific; DHN subclasses respond to drought differentially (e.g., *Trifolium repens* DHNs; Vaseva et al., 2014).

Numerous gramineous DHNs are active in drought, and some of them are family specific. Verma et al. (2017) examined the genomes of several rice species to identify upregulated DHNs under drought stress. DHNs play a crucial role in mitigating dehydration stress between various species of rice. Among the almost 65 identified DHNs, the *DHN8* gene of *O. sativa* ssp. *japonica* showed an up to 448-fold increase in expression on the first day of stress treatment. Studying the response of wheat plants (*T. aestivum*) to drought, Vassileva et al. (2012) observed that the levels of various DHNs and HSPs increased among the varieties that are more resistant to adverse conditions. Among the *Brachypodium distachyon* ecotypes, at least ten different *DHN* genes participated in the response to drought in leaves, but only four *DHN* genes appeared to be orthologous to genes from other grasses (Decena et al., 2021). In maize (*Z. mays*) and juvenile barley (*H. vulgare*) seedlings, the level of transcripts for various DHNs (e.g., DHN1 or COR410) was significantly elevated under water deficiency (Wehner et al., 2016; Zhang et al., 2020). The abundance of *O. sativa* DHN1 protein decreased in moderate and severe drought (Habibpourmehraban et al., 2022). Furthermore, numerous DHNs and other LEA proteins increased their abundance in leaves of drought-tolerant and sensitive wheat cultivars under water deficit, which were profiled using a label-free quantitative approach; almost 20 and 14 LEA proteins appeared to be involved in the development of drought tolerance in stress-tolerant and sensitive genotypes, respectively (Li et al., 2018). In other wheat cultivars, highly abundant DHNs (of 66 and 50 kDa), presumably corresponding to the WCS66 and WCS120 proteins at 50% and 40% of the soil water content, respectively (Kosová et al., 2022), decreased as the drought became milder, and interestingly, the abundance of those DHNs became more affected by invasion by aphids (*Aphidoidea*).

Other proteins, namely, ERD10-like and ERD14-like proteins, responded in abundance under drought stress in canola (*B. napus*), as indicated by Koh et al. (2015) study. Gene differential expression was investigated from the 3rd day and lasted until the 14th day of drought, and both DHNs accumulated mainly on the 10th day (in addition, ERD10-like protein appeared to be abundant on the 7th day of stress). Canola drought-responsive DHNs became part of the larger category of proteins involved in stress and defense processes, to which other LEA proteins, HSPs, peroxiredoxins, and ROS scavengers also belong. Interestingly, the intensity of phosphorylation of the S90 residue in the ERD14 canola protein (XP_013717955.1) positively correlates with the duration of drought (Wang et al., 2016). Rurek et al. (2018) also observed elevated expression of ERD14 and ERD14-like genes in the drought-sensitive ‘*Adelanto*’ cultivar of cauliflower (*B. oleracea* var. *botrytis*) under mild and severe water deficit; however, in another stress-sensitive cultivar (‘*Casper*’), severe drought decreased their transcript level. Furthermore, ERD14 and ERD14-like DHNs were also identified by mass spectrometry after 2D PAGE. As expected, genes for the ERD transcription factors became upregulated in drought-tolerant lines under long-lasting drought (Estrella-Maldonado et al., 2021). However, drought exposure of *Brassica rapa* plants resulted in a decrease in the level of the DHN family protein (Bra015779), especially under 24 h of stress (Kwon et al., 2016).

Drought acclimation has also been investigated in relation to other DHNs and dlps of organellar localization or interacting with organelles. Guo et al. (2019) studied the effects of *Saussurea involucrata* DHN (*Si*DHN) overexpression in tomato (*Solanum lycopersicum*) plants on plastid protection against negative drought effects. The highest abundance of the *Si*DHN protein was achieved after 12 hours of stress. The accumulation of DHNs under those conditions resulted in increased water, chlorophyll, and carotenoid content, as well as elevated PSII quantum efficiency. In addition, transgenic plants showed an increase in the level of ROS scavenging enzymes that provide plant resistance to secondary oxidative damage. Another example of a DHN with altered abundance in response to drought is the *Sh*DHN from *Solanum habrochaites*, which was shown to increase its abundance almost 12 times in the initial stress phase (Guo et al., 2019). Cold treatment resulted in even more pronounced (> 40-fold increase) alterations in the level of similar DHNs (Liu et al., 2015; Yu et al., 2018). Moreover, the level of other plastid DHNs—the *Pp*DHNA and *Pp*DHNC of *Physcomitrella patens*—increased during drought acclimation, which resulted in increased stress tolerance as the content of ROS scavenging enzymes also increased (Li et al., 2017). The expression of *DHN* genes in highly tolerable sunflower (*Helianthus annuus*) varieties in response to progressive water deficit also confirms the above observations (Cellier et al., 1998).

Regarding DHNs/dlps interactions with mitochondria, in drought-treated winter wheat (*T. aestivum*), rye (*Secale cereale*) and maize (*Z. mays*) seedlings, increased accumulation of dlps has been observed. They were proposed to stabilize mitochondrial membranes and other organellar proteins (Borovskii et al., 2002). Rurek et al. (2018) investigated the pattern of proteins identified by antibodies to the K-segment in mitochondria of three cultivars of cauliflower (*B. oleracea* var. *botrytis*). Small proteins of 18-37 kDa detected by K-segment-specific antisera and 30/35 kDa proteins detected by SK_3_ motif-specific antibodies increased significantly but differently in abundance in stress-sensitive cultivars in mild or severe water deficit, in contrast to drought-resistant cultivar. Czernik et al. (2020) transfected sheep fibroblasts with constructs containing cDNA for three different DHNs with diverse subcellular locations (mitochondria, cytoplasm, and cell nucleus). The tested cells were subjected to a water shortage. After restoring to unstressed conditions, most fibroblasts transformed by an empty vector did not survive under stress, while in cells overexpressing DHN, the survival rate ranged from 30% in the case of cells with *in cellulo* overproduction of a single protein to 58% in variants overexpressing all three DHNs simultaneously. In recombinant cells, no alterations were observed in the mitochondrial morphology, cell membrane, and cell cytoskeleton.

Although DHNs are particularly prevalent among plants, it should be noted that LEA proteins also participate in the drought response of other organisms. A well-known example might be *Artemia franciscana*, a crustacean (*Crustacea*) species capable of anhydrobiosis that contains proteins from the LEA family targeted to mitochondria. They are involved in the development of tolerance to extreme environmental conditions (Hand et al., 2018).

## 6 Specific functions of organellar dehydrins

**Figure 2:**
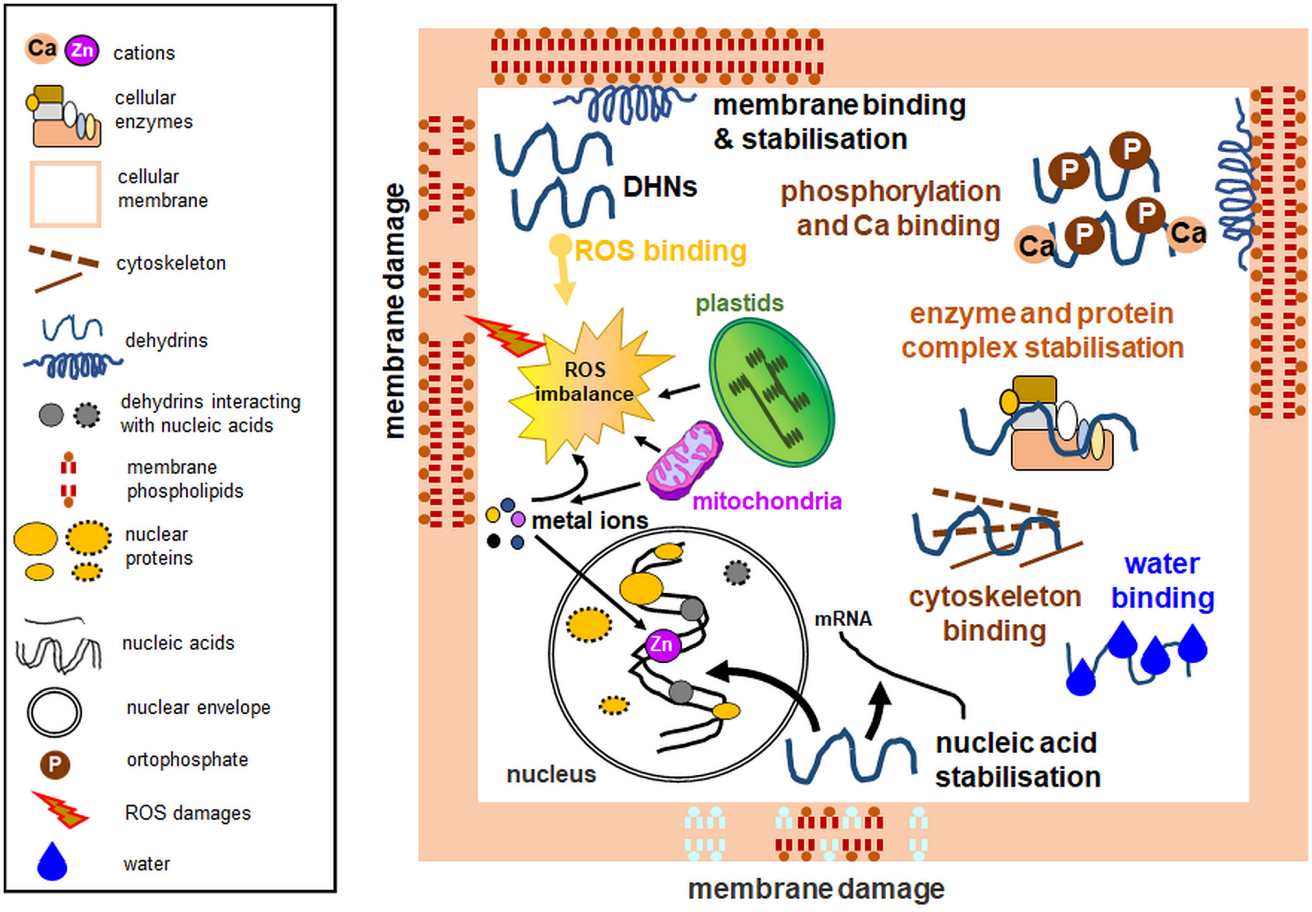
Experimentally verified dehydrin functions with emphasis on organellar protection. Among multiple activities, dehydrins lower the temperature of the membrane phase transition, prevent protein aggregation, maintain enzymatic activity, possess antioxidant properties, bind water, interact with the cytoskeleton, and protect nucleic acids. Some dehydrins do not perform all the biological functions depicted here. Diagram based on the data from multiple analyses, partly based on the concept from the data of Hara (2010). *Left*: a legend for the figure details.

The disordered structure of DHNs is thought to allow them to have many cellular functions, such as the protection of the intracellular membrane system, including organelles. DHN functions depend on their subcellular localization, protein structure, and type of stress stimuli. In subarctic-grown, cold-acclimated *Thellungiella salsuginea* species, two DHNs stabilize their own secondary structure by interaction with Zn^2+^ both freely and interacting with membranes; however, this is not a rule under cold stress for other DHNs. Generally, DHNs specifically interacting with some macromolecules will induce secondary structures. However, at low temperature and in the presence of Zn^2+^, secondary structure redistribution occurs for both studied DHNs rather than induction (Rahman et al., 2011). As mentioned in Chapter 4, DHN proteins lower the temperature of the membrane phase transition and thus protect cell organelles under various stress conditions (**Figure 2**) (Eriksson et al., 2011; Clarke et al., 2015; Andersson et al., 2020).

**Figure 3:**
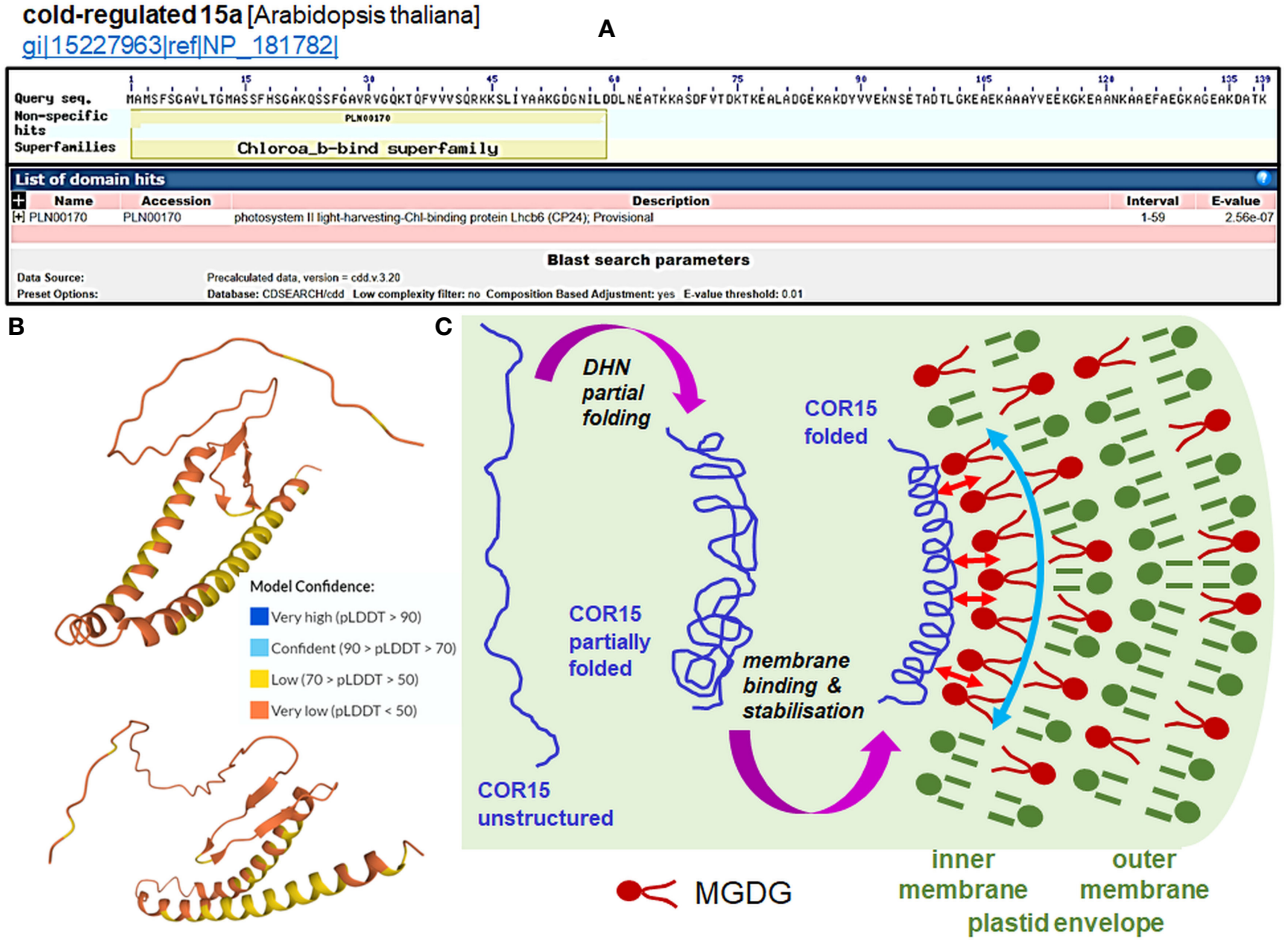
Function of the COR15 proteins (data in panels **A** and **B** for COR15a only). **(A)** Prediction of the domain of the Arabidopsis COR15a protein (NP_181782.1; all data accessed from ncbi.nlm.nih.gov/Structure/cdd/wrpsb.cgi?seqinput=NP_181782.1). **(B)** Predicted 3D model of AtCOR15a (Q42512; CR15A_ARATH UniProt record) by AlphaFold (accessed from uniprot.org/uniprotkb/Q42512/entry#structure). Exemplary projections with a particularly low per-residue confidence score (pLDDT) through all protein residues indicate a lack of apparent structure in diverse protein regions of COR15. Enhancing the stabile folding of COR15 is generated by the membrane interaction (see panel **(C)**. **(C)** Cell dehydration caused by freezing results in a closer apposition of intracellular membranes to each other and macromolecule crowding, which causes partial folding of the COR15 protein (*first magenta arrow*). Thalhammer and Hincha (2014) estimated that COR15 can occupy ca. 15% of the inner membrane of the surface of the plastid envelope. Plastid membranes contain monogalactosyl-diacylglycerol glycolipids (MGDG), which are unstable under low temperature. The COR15 protein interacts with the inner membrane of the plastid envelope enriched with MGDG (*small red arrows*), which improves the helical conformation of COR15 (*second magenta arrow*). Binding of COR15 to the MGDG-enriched plastid membrane occurs with the hydrophobic face of the amphipathic helix of COR15. This association results in enhanced folding, which exposes a larger hydrophobic face capable of interaction with membrane lipids. In addition, the helix may be stabilized on the membrane by hydrogen bonding with MGDG, which orients it parallel to the membrane surface. Overall, it provides stabilization of the membrane (*curved vertical arrow*) under freezing stress, thus preventing the formation of hexagonal phase II lipid domains. Such membrane stabilization maintains plastid integrity and functionality, as can be indicated by the electrolyte leakage test (panel **(C)** partly based on the concept from the data of Thalhammer and Hincha, 2014).

Two particular DHNs—COR15A and COR15B DHNs—protect plastids when under cold stress from electrolyte leakage by assuming specific secondary structures, allowing them to bind to the outer membrane (Thalhammer et al., 2014); alone, they are highly unstructured. **Figure 3** presents the mechanism of interactions of COR15 DHN with plastid membranes under freeze stress proposed by Thalhammer and Hincha (2014). The Arabidopsis COR15a and COR15b proteins contain an N-terminal chlorophyll *a*-*b*-binding domain, similar to the PSII light harvesting chlorophyll-binding protein, and are largely unstructured proteins in the absence of a bound membrane (**Figures 3A, B**). Plastid membranes contain monogalactosyl-diacylglycerol glycolipids (MGDG), which are unstable under stress conditions. The secondary structure of the two DHNs mentioned above enables them to bind to MGDG and provide membrane stabilization (details on the membrane binding by COR15 are shown in **Figure 3C**). Binding of DHNs to organelle membranes has also been confirmed for the *Ts*DHN-1 and *Ts*DHN-2 proteins of *Thellungiella salsuginea*. *Ts*DHN-1 binds to the membrane at 4°C by hydrogen bonds, stabilizing the lipid monolayer. On the contrary, *Ts*DHN-2 interacts with the membrane at reduced temperature, using electrostatic interactions, and participates in the maintenance of its structure (Rahman et al., 2011; Rahman et al., 2013).

Diverse stress conditions often result in oxidative burst, which develops mainly in chloroplasts and mitochondria, where electron leakage and ROS formation occur. ROS pose a threat to the intracellular membrane continuum (Juan et al., 2021). Cell protection against ROS can also be provided by DHNs, with attributed antioxidant properties (**Figure 2**), due to the high content of amino acid residues such as glycine, histidine, and lysine, which are targets in ROS-mediated protein oxidation (Sun and Lin, 2010). *Cu*COR19 DHN from *Citrus unshiu* mitochondria treated at low temperature showed an inhibitory effect on membrane peroxidation, which was stronger than other antioxidants used (Hara et al., 2003). ROS scavenging has also been attributed to *Pp*DHNA and *Pp*DHNC proteins of *Physcomitrella patens* (Li et al., 2017). Protection of cells from adverse ROS effects can be achieved through the interaction of DHN with heavy metals. Metal atoms, because of stress, can be a source of ROS. Therefore, metal binding by DHN can prevent ROS formation and protect cellular structures. In addition, histidine-rich regions are thought to be responsible for DHN interactions with metals (Hara et al., 2005). To investigate the ability of DHN to bind metals, Hara et al. (2005) studied *Cu*COR15 interactions with various divalent metals and confirmed the highest affinity of the protein for Cu^2+^ and the involvement of histidine-rich motifs in metal ion binding.

Due to their high content of polar amino acids, DHNs can also bind water molecules (**Figure 2**). This interaction prevents water loss and allows regulation of the ion concentration as it increases in drought (Tompa et al., 2006). DHNs can bind to partially dehydrated regions of other proteins and thus protect them from denaturation (Drira et al., 2013; Drira et al., 2015; Yang et al., 2019b). Water retention during drought also serves to stabilize proteins and cellular compartments (Close, 1996; Shekhawat et al., 2011; Liu et al., 2015). Furthermore, some DHNs bind Ca^2+^ ions, as noted for acidic Arabidopsis DHNs including ERD14, ERD10, and COR47 proteins. Ca^2+^ binding can often occur simultaneously with DHN phosphorylation (**Figure 2**). Therefore, Alsheikh et al. (2005) proposed that DHN phosphorylation occurs first, thus allowing subsequent Ca^2+^ ion binding.

DHN multifunctionality also includes their interactions with the cytoskeleton (**Figure 2**). ERD10 DHNs have been shown to bind to actin filaments and thus participate in the inhibition of actin polymerization (Abu-Abied et al., 2006). Another important role for DHNs is nucleic acid protection (**Figure 2**; Boddington and Graether, 2019). An experiment conducted on the *Cu*COR15 protein showed that it bound DNA in the presence of Zn^2+^ ions (Hara et al., 2005). In the electrophoretic mobility shift assay (EMSA), Y_2_K DHN of bean (*Vigna radiata*) bound only shorter DNA fragments (3 to 4 kb) non-specifically without ion addition; furthermore, ion binding stimulated DNA binding, and some ions (e.g., Ni^2+^) enhanced DHN binding with shorter DNA fragments (0,7-1 kb; Lin et al., 2012).

DHNs can also protect organelles from freezing and subsequent thawing by preventing membrane fusion (Clarke et al., 2015), protein aggregation, and decreased enzyme activities (**Figure 2**; Hughes et al., 2013). Multiple studies characterizing various DHNs (including those from plant biomass, e.g., Liu et al., 2022) employed known enzymes (for instance, LDH) for *in vitro* tests. Oxidative damage often accompanies the stress action, and DHNs have also been shown to protect LDH against hydroxyl radicals (Halder et al., 2018). *In vitro* analyses of PCA60 DHN examined the responses of the protein subjected to freezing and subsequent thawing stress. Analysis of ice crystals and retained LDH activity indicated the antifreeze properties of the PCA60 protein (Wisniewski et al., 1999). Cryoprotective properties were also shown, for instance, by the interaction of COR15A with the chloroplast envelope (Steponkus et al., 1998). Bozovic et al. (2013) identified a few DHNs (ERD14, LTI29, and COR47) that bind to thylakoid membranes under freezing stress and exhibit cryoprotective effects. Szabała (2023) also showed the cryoprotective activity of purified DHN24 in the enzymatic assay and inhibition of bacterial growth under DHN24 overexpression. Interestingly, wheat DHN-5 showed weaker LDH protective effects than the heat-stable protein fraction from *Opuntia ficus-indica* seeds (Drira et al., 2013; Drira et al., 2023). Some vacuolar Ca^2+^-binding proteins also showed cryoprotective activity against LDH (Osuda et al., 2021).

The cryoprotection of enzymes can be shown comparably both by the K- and F-segments and depends on hydrophobic residues (Ohkubo et al., 2020); however, in the Arabidopsis HIRD11 protein, its disordered structure, the K-segments, and the non-K-segment 1 showed cryoprotective characteristics by inhibiting PC liposome aggregation even better than cryoprotective agents (Yokoyama et al., 2020; Kimura et al., 2022). Hughes et al. (2013) showed that the number of K-segments affects the hydrodynamic radius of DHNs and the LDH protection activity and that the intrinsic disorder of DHNs is necessary for their cryoprotective properties. Hydrophobic residues of the K-segment participate in the prevention of the cryoinactivation, cryoaggregation, and cryodenaturation of LDH (Hara et al., 2017).

Recently, Smith and Graether (2022) proposed a different mode of cryoprotection for *Vitis riparia* YSK_2_ dehydrin towards the cold-labile yeast frataxin homolog-1 (Yfh1); it relies on presumably weak interactions employing positively charged residues within the YSK_2_ sequence that keep the helical structure of Yfh1 at a low temperature. In general, it is different from the LDH protection model, which is primarily dependent on the hydrodynamic properties of the enzyme. The results of the Smith and Graether (2022) study thus support the idea that DHNs may show cryoprotective activities by multiple mechanisms.

## 7 Dehydrin-like proteins

### 7.1 Controversies on the categorization of dehydrin-like proteins

*Bona fide* DHNs in stress response are often accompanied by proteins referred to in the literature as dehydrin-like proteins (dlps) and dehydrin-related proteins (drps). Dlps and drps are often identified with antibodies against the K-segment (Close, 1997), which may reflect their immunological similarity to DHNs. Therefore, they should be described as proteins identified by antisera specific to DHN segments rather than dlps; their further relationship with DHNs is unclear as dlps are still poorly characterized. For clarity, we will generally refer to these proteins as ‘dlps’ or ‘drps’; however, in discussing respective literature data, we will cite them according to the state of knowledge.

SDS-PAGE and size exclusion chromatography are often used to determine the size of DHNs and related proteins. However, it should be noted that the methodology for determining the IDP size is unreliable. According to Receveur-Bréchot et al. (2005), differences in mass measurements due to the IDP structure can overestimate their size by up to two times. The lack of internal organization by DHNs and dlps results in weaker binding of the protein to the SDS, resulting in the slower migration of the protein on the gel (Receveur-Bréchot et al., 2005; Haider et al., 2012). Exclusion chromatography, on the other hand, involves separating biomolecules of different sizes using porous gels. Determining the size of proteins involves measuring the column retention time, which increases with IDPs (Kostanski et al., 2004; Receveur-Bréchot et al., 2005). Thus, some reports propose additional criteria to distinguish DHNs from dlps. According to Rurek (2010), protein thermostability should also be taken into account. ‘True’ DHNs are resistant to high temperatures and remain soluble after boiling (10 min at 100°C; Shi et al., 2016). Therefore, DHNs remain heat stable and cannot be denatured, thus allowing them to be purified from other proteins without additional chromatographic purification or protease inhibitors (Livernois et al., 2009). On the contrary, numerous dlps and drps are significantly more thermolabile (Borovskii et al., 2002; Rurek, 2010). Some data on non-plant proteins related to DHNs are also known. For example, Pochon et al. (2013) described a conserved DPR motif of asparagine, proline, and arginine residues present in fungal DHNs and usually occurring in a few repeats. These authors, after *in silico* genomic search, found three proteins similar to DHNs in a fungus *Alternaria brassicicola*, which contained similar motifs.

### 7.2 Subcellular localization and interactions

It is worth paying attention to the subcellular location of dlps, which may suggest their function. A study by Rurek (2010) on the accumulation of cauliflower (*B. oleracea* var. *botrytis*), Arabidopsis, and yellow lupine (*L. luteus*) proteins, identified by K-segment specific antibodies under various conditions of abiotic stress (cold, heat, and freezing), revealed that most of the identified proteins were distinct from other Brassicacean DHNs and dlps by size (Battaglia et al., 2008; Bies-Ethève et al., 2008) and were interacting with mitochondrial membranes. Cauliflower thermolabile proteins identified by antibodies to DHN segments were found mainly in the mitochondrial matrix, indicating their active import into the organellar lumen. Here, for their detection, two independent antisera were used, thus minimizing cross-reactive results. Due to the relatively different amounts of K-segments or similar motifs in different dlps, or to the non-equal number of accessible epitopes for dlps recognized by those antibodies, results obtained with K-segment-specific antibodies and for the SK_3_-type of DHNs differed substantially. Stress appeared to affect not only the relative level of proteins identified by antibodies to DHN segments but also the number of immunoreactive epitopes of particular proteins (Rurek, 2010). However, in wheat (*T. aestivum*), the presence of other proteins identified by antibodies to the K-segment was observed under cold stress; these proteins were associated with the outer mitochondrial membrane (Borovskii et al., 2005).

**Figure 4:**
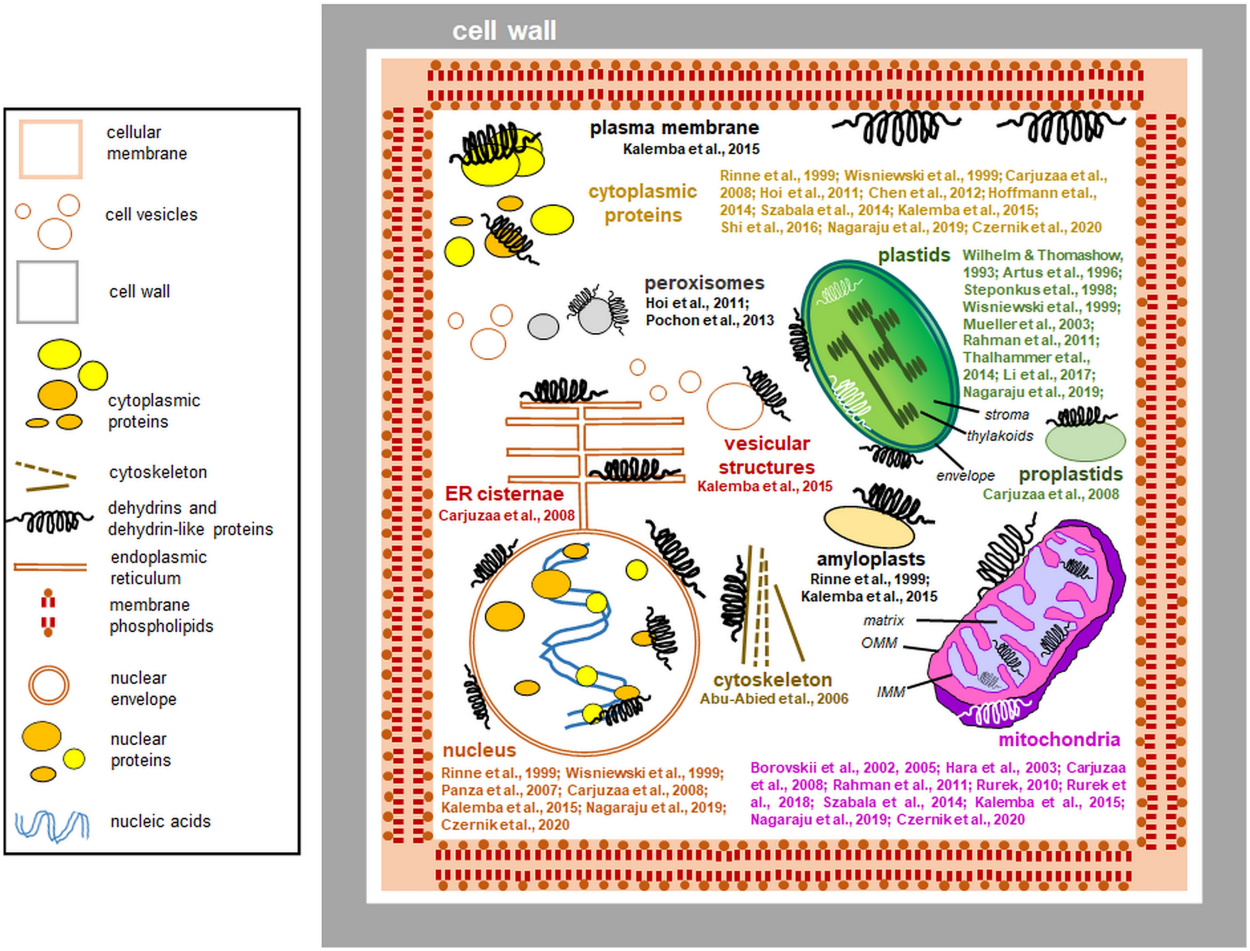
Interactions of dehydrins and dehydrin-like proteins (including proteins detected by antibodies against DHN segments) with plant cell compartments (the data compiled from the literature and references to studies of particular proteins are indicated). The most important references are shown close to each compartment depicted. Most dehydrins protect organelles from outside; instead, proteins immunologically related to dehydrins are found also in the organellar lumen. The proteins are drawn schematically, and their sizes are disproportionately enlarged. *Left*: a legend for the figure details. Details of the plastid and mitochondrial ultrastructure are also shown: IMM, inner mitochondrial membrane; OMM, outer mitochondrial membrane.

Dlps have been shown to interact with other organelles. Carjuzaa et al. (2008) studied the accumulation of K-segment antibodies in *Chenopodium quinoa* embryos. Antibodies targeting the K motif were used, and the size of the detected proteins was compared with that of known wheat DHNs. Surprisingly, the only single protein showed similarity to previously characterized DHNs. Furthermore, by immunogold labeling, it was possible to determine that antibodies identified by the K-segment are present in the cytoplasm and interact with the membranes of the endoplasmic reticulum, the inner membrane of mitochondria, and even the envelope of proplastids. They have also been localized in the cell nucleus, where they interact with euchromatin (Carjuzaa et al., 2008). Nuclear localization and interactions of proteins identified by antibodies to the K-segment with chromatin have also been confirmed for proteins from mature *Euterpe* seeds (Panza et al., 2007). Kalemba et al. (2015) compared the accumulation and subcellular localization of three distinct proteins identified by antibodies to the K-segment of beech (*Fagus sylvaticus*) embryos derived from seeds stored for two years. In seeds during maturation between the 12 and 19 weeks after flowering, dlps were localized in the interior of the nucleus, cytosol, membrane vesicles, and around the cell membrane. Other dlps were also associated with the mitochondrial envelope and amyloplast membranes. In biennial seed cells, the levels of dlps were much lower, and they were associated with vacuolar and nuclear membranes and localized inside the amyloplasts. Hoi et al. (2011) showed that BprA and DprB dlps accumulate in the cytoplasm and peroxisomes of *Aspergillus fumigatus* hyphae cells. Furthermore, *Ab*Dhn1 and *Ab*Dhn2, proteins similar to DHNs in *Alternaria brassicicola* mycelium, were shown to interact with peroxisomes (Pochon et al., 2013). **Figure 4** summarizes the data on the subcellular location of plant dlps (including especially proteins identified by antibodies specific to the DHN segments) based on the known data.

### 7.3 Current perspectives on participation of dehydrin-like proteins in plant development and stress acclimation

Like DHNs, dlps can also affect stress acclimation. Plant conditioning has traditionally been known to improve germination performance (Sen et al., 2021). Dlps can be involved in osmopriming, which involves the hydration of seeds to stimulate their metabolic activation and in parallel does not initiate embryo development (Kubala et al., 2013). In *Ranunculus sceleratus* seeds, it has been shown that during this process, among other macromolecules, the abundance of proteins identified by antibodies to the K-segment increases (Wechsberg et al., 1994). To better understand the function of dlps in seed conditioning, Chen et al. (2012) studied the germination strength of spinach (*Spinacia oleracea*) seeds under stress conditions. They observed that proteins, identified by antibodies to the K-segment, participate in the first stages of seed priming. They then studied seed germination under drought conditions and observed elevated accumulation of those proteins in plants grown from conditioned seeds and a greater tolerance of seed embryos to drought.

Dlps are also involved in cold acclimation. In a study on cold tolerance in perennial grasses, changes in dlp accumulation were observed. Analyses were conducted on creeping bentgrass (*Agrostis stolonifera*) and two annual panicle cultivars (*Poa annua*), one of which was characterized by increased sensitivity to cold and the other resistance to it. The plants were subjected to low temperature, and the temperature was subsequently increased to induce deacclimation. Analysis of differences in dlp accumulation allowed us to characterize the dlp of 18 kDa in *A. stolonifera* and of 40 kDa in two annual bluegrass ecotypes, which differed in accumulation level during the acclimation and deacclimation. Together with the other factors studied, dlp was responsible for differences in cold tolerance (Hoffman et al., 2014). Tolerance to low temperatures was also studied in the *Ammopiptanthus mongolicus Am*CIP protein, which exhibited DHN characteristics. *A. mongolicus* belongs to highly tolerant species at low temperatures and is found only in the Alashan Desert, where during winter temperatures drop to -30°C. *Am*CIP overproduction was carried out in *E. coli*; after cold stress, the recombinant strain acquired a notable stress tolerance. The cryoprotective properties of the *Am*CIP protein against the bacterial LDH activity were shown, and the elevated level of *Am*CIP also increased the resistance to freezing. Furthermore, overexpressed *AmCIP in planta* in tobacco (*N. tabacum*) increased cold tolerance during seed germination and seedling growth (Shi et al., 2016). *In planta*, overexpression of another DHN from *Ammopiptanthus nannus*, a related rare legume species from the desert of China, allowed an increase in the germination rate, peroxidase and catalase activities, a higher content of water and proline, and a decrease in malondialdehyde level. Furthermore, the Arabidopsis root phenotype was altered (Sun et al., 2021a). Rinne et al. (1999) characterized ABA-responsive DHN homologs present in birch (*Betula pubescens*) meristem cells. In this tissue, which was not exposed to stress, only a single type of cytoplasmic protein was identified. However, after cold acclimation, the accumulation of two additional proteins of this type was observed and localized, inter alia, in amyloplasts and in the nucleus. This observation indicates that some drps can be relocated in response to stress within plant cells.

Changes in the accumulation of proteins identified by antibodies to the K-segment under cold, freezing, and heat have been shown in yellow lupine (*L. luteus*), cauliflower (*B. oleracea* var. *botrytis*), and Arabidopsis mitochondria (Rurek, 2010). Under cold treatment, the abundance of low molecular proteins decreased both in lupin-imbibed seeds and hypocotyls (proteins of 25-40 kDa were good candidates for cold-induced dlps in lupin mitochondria that are probably distinct from other known ones in this genus [Pinheiro et al., 2008]), while the level of accumulation of high molecular proteins did not change. However, increased temperature caused an increase in the abundance of several other proteins (20 to 40 kDa in hypocotyls and 30-40 kDa in seeds) with a putative mitochondrial location; in cauliflower and Arabidopsis dlps, variations in abundance under heat were more pronounced (Rurek, 2010). Therefore, all the mentioned proteins are involved in protecting cell compartments from adverse stress effects.

## 8 Conclusions and future directions

DHNs, which allow plant resistance to unfavorable stimuli, can be seen as the ultimate protein effectors responsible for prompt phenotypic changes in stress (Yadav et al., 2023). Because of their disordered structure, DHNs interact with various molecules, including membrane lipids, allowing the stabilization of organelle membranes and protecting them against adverse conditions. The protective status of DHNs also includes some other functions: these proteins can protect cells from ROS damage, stabilize DNA and enzymes, and affect cell biogenesis in numerous ways. With the progressive research on the structure of these proteins, we have learnt more and more about the mechanisms of their interactions; however, a few points regarding DHN characterization should be elucidated soon.

First, the cellular functions of recently described DHN subtypes, including F_n_K_m_ and F_n_SK_m_ proteins, and their subcellular localization should be further investigated. Additionally, it would be revealing to find more orthologs of plant DHNs/dlps in other taxa, including fungi, animals, or even unicellular organisms. Organelle functions are important in the development of tolerance to selected stress conditions, especially to cold (Maryan et al., 2021); therefore, the participation of DHNs/dlps in organellar biogenesis should be investigated more intensively. Furthermore, data on organellar DHNs/dlps should more broadly encompass agronomically important species as this knowledge can potentially be used in the future to construct novel or genetically modified crop genotypes that are resistant to adverse environmental stimuli, especially because systemic analyses have been deepened recently for some of these species, including *Brassica* (Yadav et al., 2023).

Challenges with dlp categorization indicate that it is necessary to find more reliable criteria for it and to characterize dlps in more detail. In parallel, the cloning of genes/cDNAs for most dlps and detailed tissue and organ-specific expression profiles of dlps must be investigated in detail. Special attention should be paid to elucidating the expression of DHNs/dlps in floral tissues and the potential role of these proteins in the biogenesis of certain genetic traits, including pollen sterility. This goal may be achieved relatively easily as complete transcriptomic and proteomic data of numerous plant species (including model ones) are already available.

However, not much is known about the interactions of DHNs with other organellar proteins and genomes, and detailed interactomic analyses employing DHNs/dlps should thus be performed in order to reveal the protein partners interacting with DHNs/dlps. In the near future, the expression patterns of DHNs/dlps should be tested in a plethora of abiotic and biotic stresses in a more systemic scale; however, focusing those investigations on the most detrimental conditions in agriculture, including drought, may be especially conducive to increasing crop productivity as drought adaptation is a particularly complex phenomenon (Salekdeh et al., 2009). It would also be worthwhile to further study the interconnections between melatonin treatment and the DHNs/dlps level. This concept raises the possibility of the construction of transgenic crop plants with *in planta* overexpression of DHNs that can be grown in the presence of exogenously sprayed melatonin (Bajwa et al., 2014). The participation of dlps in non-temperature treatments has been largely less investigated; therefore, this field should be exhaustively broadened.

Finally, detailed elucidation of the less studied DHN functions in plant cells should continue. In particular, novel ideas on the participation of new organellar DHNs and dlps in stress acclimation and deacclimation should be investigated soon using more intensive omics tools, thus broadening our knowledge on the fascinating adaptability of these proteins to numerous adverse conditions. Such data are especially important in view of the changing and affecting climate conditions of agronomically important species. Therefore, the broadened characterization of DHNs and dlps represents a vibrant and important field in plant research that may be used in the future for the successful development of stress-resistant lines/genotypes (Zhang et al., 2019). For example, recent studies such as those by Ganguly et al. (2020), who show the increased tolerance of transgenic rice (*O. sativa*) to drought under overexpression of the Rab16A protein, and Aduse Poku et al. (2020), who demonstrate the engineering of the LEA-S protein of watermelon (*Cucumis melo*) in drought and salinity, are representative examples. Another excellent study provides Arabidopsis Rab18 DHN (AT5G66400) and a few other LEA proteins, which have been shown to be affected in abundance under triple stress treatments and are therefore good candidates for stress markers (Sewelam et al., 2020). However, multiple and effective attempts to use DHN in plant agriculture should also include other methodological tools, including genomics-assisted breeding, plant gene editing, and plant metabolic engineering.

## Author contributions

ZS partially deposited and analyzed the literature data, prepared **Table 1**, and partially wrote the manuscript. MR designed the general concept of the review, partially wrote the manuscript and analyzed the literature data, prepared all the figures, reviewed the data quality, and updated the whole manuscript for publication. Both authors have accepted the final version of the manuscript and have agreed to be responsible for all aspects of the work.
